# Rush Medical College Clinic

**Published:** 1877-08

**Authors:** Edmund M. Landis


### RUSH MEDICAL COLLEGE CLINIC.

Service of PROF. MOSES GUNN.

(Reported by EDMUND M. LANDIS, M. D.)

*Pulpy Degeneration of the Knee Joint.*

Mrs. M. D., American, aged 25. The patient, whose previous health had been good, states that three years ago, she fell from a fence, alighting on her heel, after which the right knee became swollen and painful. Since then it has gradually and slowly enlarged. The knee has been more swollen than now, but has never been red. The pain is moderate but is greater than formerly. The leg is slightly flexed. The patients health has gradually failed, having lost some forty pounds.

On examination, taking the sound limb as a standard of comparison, find that the tissues on either side of the patella are larger, giving to the touch a sensation of *elasticity.*

This last fact is important, as the symptoms in the case simulate hydrarthrosis, but differ in the point of elasticity, the absence of fluctuation, and in the impression on the patient's health.

Pronounce it "Pulpy Degeneration of the Knee Joint" which has invaded the tissues about the joint, filling them up and extending outside.

The patient has come for advice as to the removal of the leg. She has been under competent treatment, but has not improved. Personal experience with this form of disease does not allow me to extend hope of benefit from palliative treatment.

Hueter claims fine results in these cases from injections of weak solutions of carbolic acid; but in the cases which have come under my observation, this has not been corroborated (the fact however, has been demonstrated, that weak solutions of carbolic acid can be injected into the knee joint with perfect impunity, as the point of the syringe was carried on a number of occasions right beneath the patella).

*Periostitis.*

John H., Irish, aged fifteen, painter. The patient is a poorly nourished lad, who, his parents state, has no hereditary taint, and whose only previous sickness was scarlet fever, which he had seven years ago.

One and a half years ago, the boy received a blow on the left tibia from a shinney stick, but at the time was not apparently much injured. Three months afterwards he was taken with severe pains in the leg, which lasted some two or three months, and which were worse at night, keeping the patient awake until two or three o'clock in the morning.

There have been five or six such attacks, with intervals of two or three months. Is suffering from one now, which commenced three weeks ago. The leg has gradually enlarged from the first, but only to a slight degree of late.

On examination find the left tibia enlarged forward along the whole extent of the crest, thus increasing its antero-posterior diameter. There is a slight, lateral enlargement, but it is confined to its lower extremity. Strong percussion elicits pain, which is greatest near the ankle.

This is a rare form of periostitis, rare in the shape of the enlargement it has produced. A healthy lad, free from disease except the attack of scarlet fever seven years previous, receives a blow on the skin, which was not painful, when three months afterwards it began to pain and enlarge. (The pain at the ankle is probably sympathetic.) We frequently encounter these enlargements, but not in this form. On grasping the tibia it is found that it is not enlarged laterally except at its lower part. The question, however, is whether we have hypertrophy or abscess. We frequently have enlargements from bone abscesses, but they increase the diameters in all directions, while the enlargement is more localized. Hypertrophy with abscess, also frequently occurs, and is also more circumscribed than in the present case. Periostitis with hypertrophy, is not usually so painful as abscess, having painful periods with exacerbations. The pains in this case have been nocturnal for two months at a time.

Have trephined for three cases, at times removing large deposits of pus, at others only obtaining a drop or two, and again not demonstrating that there was an abscess, yet in all giving relief.

Think that the nocturnal pains point to an abscess, but it would be difficult to find the point in the bone to strike it, although there are several which seem suspicious.

It is thought that an incision through the periosteum might afford relief.

The patient was anæsthetized, and an incision about eight inches in length made through the integument, along the crest of the tibia, down to the periosteum, which was then incised by five parallel incisions co-extensively with the incision in the integument.

As the incisions were made in the periosteum, some half a dozen or more enlarged periosteal vessels spurted very freely, showing its abnormally vascular condition. The edges of the incisions gaped and the periosteum was found thickened and spongy. After hemorrhage had ceased, the wound in the integument was brought together and secured by interrupted sutures.

Warm water dressings were ordered. The pain which had been regular up to the time of the operation, ceased, and has not yet returned, the patient sleeping the first night and every night since. The wound healed kindly and rapidly, and the patient has resumed his occupation.

